# Food Changes and Geography: Dietary Transition in Colombia

**DOI:** 10.5334/aogh.1643

**Published:** 2019-03-05

**Authors:** Doris Cristina Quintero-Lesmes, Oscar F. Herran

**Affiliations:** 1 Fundación Cardiovascular de Colombia, Floridablanca, South América, CO; 2 Escuela de Nutrición y Dietética, Universidad Industrial de Santander, Bucaramanga, CO

## Abstract

**Background:**

The food transition can no longer be studied in developed countries because the so-called Western diet now predominates in these areas. However, in developing countries, it is still possible to study the food transition. It is a novel concept that complements other transitions such as the demographic, economic, nutritional and epidemiological transitions.

**Objectives:**

The objectives of this study were to a) estimate the average departmental adherence to the three pre-established food patterns, b) assess adherence patterns based on the Global Spatial Analysis, c) evaluate whether the Local Spatial Variations in the adherence to food patterns are random or follow defined patterns (cluster) and d) generate 2D maps to graphically locate the food patterns that compose the phenomenon of the food transition occurring in Colombia.

**Methods:**

The National Survey of the Nutritional Situation in Colombia, 2010 was analyzed. Based on factor analysis, three consumption patterns were established; Protein/Fiber, Snack and Snack and Traditional/Starch and the average departmental adhesion was estimated. The global and local spatial variation was calculated with the Moran indexes.

**Findings:**

the average adherence to the traditional consumption/starch pattern was –0.00 (95% CI: –0.12 to 0.12). The mean adherence to the protein/fiber intake pattern was –0.07 (95% CI: –0.16 to 0.03). The average adherence to the pattern of snack consumption was –0.03 (95% CI: –0.11 to 0.05). The three patterns of food consumption values for the Global Total Moran Index, for men and women were positive and statistically significant.

**Conclusions:**

The food transition experienced by Colombia is not homogeneous and there are well defined clusters for adherence in the three predefined food patterns. Within the clusters there are differences by sex. In regions where the traditional pattern/starch predominates, the presence of the snack pattern is very weak.

## Introduction

When cultural and ecological conditions, i.e., the habitat, of households are relatively constant, the food habits and practices of the subjects do not change, or they change very slowly [1]. Among other aspects, the market economy, globalization, advertising and social pressure make subjects and households pursue new standards of living, favoring rapid and even drastic changes in food habits and practices. These changes depend on the level of social and economic development and are more evident in urban individuals and households [2,3,4,5,6,7,8]. The pattern of food consumption of a society is associated with its level of development [9,10,11]. In societies with more economic and structural development, a Western pattern predominates. In developing societies, such as Colombia, these patterns are shifting from traditional food patterns to other patterns particular to an individual’s culture or to the so-called Western culture [6,7 12,13]. Colombia is a medium- or even high-income country according to the World Bank [14]. However, it has the second-greatest economic inequality on the American continents; the Gini coefficient was 0.58 in 2018 [15]. Furthermore, Colombia is a country of regions, and the peripheral regions further from the country’s capital of Bogota, such as the Pacific and Southeast regions, display more poverty and inequality. Colombia is currently experiencing a food transition with an increase in overweight individuals and a double nutritional burden; the primary cause of death is chronic events, such as cardiovascular events and cancer [2,3,4,5,6,7,8,9,10,11,12,13]. The population changes that occur in food consumption are called food transitions. This change and others that are well described and defined, including demographic, epidemiological, economic and nutritional changes, are plausible explanations for the origin of and increase in chronic noncommunicable diseases [2,3,4,5,6,7,8,9,10,11,12,13].

Food and nutrition education, along with population interventions in a country with deep structural, cultural and economic regional differences, requires detailed information to be successful.

Based on the National Survey of the Nutritional Situation in Colombia, 2010 (ENSIN-2010), three food patterns were identified to exist in Colombia, including traditional/starch, fruit-vegetable/dairy and snack. Moreover, the food transition in this country is associated with overweight in children and adults [6,7]. Colombia is divided into 33 geodemographic units (Departments), and several of these units compose a region. Marked structural, cultural and economic differences are noted between regions. Therefore, it is valid to assume that the food transition and adherence to food patterns differ between regions [16], as previously demonstrated for the nutritional transition [8].

Although information is available regarding the food transition and its relationship with social and economic variables, its spatial distribution is unknown. A spatial analysis allows the identification of information on food patterns in two or more dimensions, thereby permitting visualization of this information on maps [17,18]. The basic concept of spatial analysis is spatial dependency or autocorrelation, analyzing the lack of independence that occurs between the observations of a variable, i.e., adherence to food patterns, for its different locations, i.e., the geodemographic units [18,19]. Spatial correlation can be positive or negative and is determined by the Moran Index, which establishes two dimensions: global and local. The Moran Global Index (Moran I) can assume positive and negative values depending on the sign of the spatial correlation. The Moran I has a normal asymptotic distribution and a first order defined by the common borders in the geodemographic units. Thus, this index is provided in a general manner for the entire surface. The statistical significance of the spatial autocorrelation can be established using a test. This index is interpreted in a similar manner to Pearson’s r correlation coefficient, providing a linear correlation. The autocorrelation may not occur on the entire surface but only in certain areas or points. Thus, it is necessary to resort to the so-called local indicators of spatial association, the Moran Local Index or LISA (Local Indicators of Spatial Association), to establish whether the statistic obtained for each zone provides information about the relevance of similar values around it [18,19].

The objectives of this study were to a) estimate the average departmental adherence to the three pre-established food patterns, b) assess adherence patterns based on the Global Spatial Analysis, c) evaluate whether the Local Spatial Variations in the adherence to food patterns are random or follow defined patterns (cluster) and d) generate 2D maps to graphically locate the food patterns that compose the phenomenon of the food transition occurring in Colombia.

## Materials and Methods

Colombia is located in the northeast corner of South America. It is a developing country, and deep economic, structural development and sociocultural inequalities are noted within its regions [20,21,22].

During 2017, an ecological study was performed based on population data collected in the ENSIN-2010. The ENSIN-2010 was performed by the Colombian Family Welfare Institute (Instituto Colombiano de Bienestar Familiar – ICBF), and the methodology was previously published [23]. In summary, the participants were selected to represent 99% of the population through stratified sampling in multiple stages. All municipalities from the 33 geodemographic units of the country were grouped into strata with similar sociodemographic characteristics. The strata were represented by randomly selected municipalities, maintaining a probability proportional to the size of the stratum. In each stratum, clusters of 10 randomly selected households were formed. The members of the household were invited to participate. The survey included 50,670 households.

### Estimation of mean departmental adherence to food patterns

The ENSIN-2010 is estimated through a food frequency questionnaire (FFQ) using ten response categories for the last month, frequency of consumption of 30 foods or food groups and three related practices. In the ENSIN-2010, the FFQ was applied in a subsample of 17,897 subjects between 5 and 64 years old [23]. For the present analysis, we chose adults between 18 and 64 years old (n = 7138), excluding pregnant women (n = 41). In addition, since it is well known that subjects at the extremes of the nutritional status spectrum overreport or underreport their dietary intake [24,25], to avoid information bias in the FFQ, the analysis was limited to subjects with a height > 100 cm and <200 cm and a weight ≥ 40 kg and <200 kg. The subsample ultimately analyzed 5217 subjects. The FFQ was applied via direct interview by nutritionists and dietitians, and the answers were recorded directly on portable digital assistants. The ICBF obtained informed consent from participants prior to their enrollment [23].

The details of the estimation of adherence scores were previously published [6,7]. In summary, the FFQ response categories were converted into a continuous variable (frequency/day) using appropriate denominators to express the frequency of consumption in units of time, “day” [26,27,28]. Based on the factor analysis, three food patterns were identified from food items and their respective frequency/day of consumption. Then, adhesion scores (Z) were established for each factor based on the factor loads and the frequency/day of the items that compose it. Finally, the mean adherence to the food patterns in each geodemographic unit was calculated as the average of the scores and total and based on sex. The details of the factorial analysis and the goodness of fit of the factorial model can be requested from the authors. The three established patterns were fruit-vegetable/dairy [*milk, cheese, kumis, yogurt, cream cheese …, raw vegetables, cooked vegetables, whole fruits, fruits in juice, bread, arepa or cookies, whole-grain, chicken, black pudding or beef viscera, low calorie foods (light), tuna or sardines*], traditional/starch [*panela, sugar, honey, rice or pasta, fried foods, dry beans, tubers or banana, eggs, beef, veal, pork …, fish or seafood, coffee or tea*] and snack [*package foods, sweets, soft drinks (powder, box, bottle), fast food, butter, sausages, chicken starters*]. The factor loads for each food within the pattern are presented in Table 1. Dietary intake has traditionally been studied at several levels: a) nutrients, b) food and c) consumption patterns. An individual generally consumes all three patterns because they are complementary to one another and constitute the total intake. By using Z scores to measure adherence to each pattern, we can identify which pattern is predominant in a subject. The same situation occurs at the ecological level, such as in this study. In each studied geographical region, all three patterns occur because they are complementary. However, one pattern predominates in each region.

**Table 1:** Loading factors (L) of foods in each pattern, Colombia, 2010.

Pattern/Ítems	L

Fruit/Vegetable/Dairy	

Milk (liquid or powder)	0.41
Cheese, kumis, yogurt, cream cheese …	0.41
Raw vegetables	0.46
Cooked vegetables	0.41
Whole fruits	0.43
Fruits in juice	0.53
Bread, Arepa or Cookies	0.25
Whole-grain foods	0.35
Chicken	0.25
Black pudding or beef viscera	0.17
Low-calorie foods (light)	0.25
Tuna or sardines	0.13
**Snack**	

Packaged foods	0.46
Sweets	0.40
Soft drinks or soft drinks (powder, box, bottle)	0.47
Fast food	0.42
Butter, cream, butter	0.11
Sausages	0.44
Chicken giblets	0.08
**Traditional/starch**	

Panela, sugar, honey	047
Rice or pasta	0.46
Fried foods	0.43
Dry beans	0.33
Tubers or banana	0.27
Eggs	0.33
Beef, veal, pork …	0.31
Fish or seafood	–0.17
Coffee or tea	0.21

### Global spatial analysis

The sample size for this analysis was 33, which corresponded to the existing geodemographic units in Colombia. The first step of the analysis was to calculate the global spatial variation in food patterns. A weight matrix was constructed with each of the adherence estimates of the three pre-established food patterns to specify the spatial relationships of the units of study (departments), such that units closer in space have greater weight than units further away. The Moran I was used as an indicator in this aspect, and the spatial autocorrelation was determined, which evaluated the degree of similarity observed between a certain location and its neighboring units [18]. The global detection given by the slope of the line of the comparison between the values of the variable considered (food patterns) for a spatial unit and that of its neighbors was estimated as follows:

I\,\, = \,\,\,\frac{n}{{\sum\limits_{i = 1}^{i = n} {\,\,\,\,\,\,\,\,\,\,\sum\limits_{j = 1}^{j = n} {{W_{ij}}} } }}.\frac{{\sum\limits_{i = 1}^{i = n} {\sum\limits_{j = 1}^{j = n} {{W_{ij}}} } \left( {{x_i} - \bar x} \right)\left( {{x_j} - \bar x} \right)}}{{\sum\limits_{i = 1}^{i = n} {{{\left( {{x_i} - \bar x} \right)}^2}} }},

where *n* is the number of units in the map, *W_ij_* the distance matrix that defines whether the units are neighbors, X_i_ is the value of the adherence to each of the three patterns in each geodemographic unit, and Z is the mean of the variable X. The index is normalized by subtracting its mean value and dividing the difference by its standard deviation, the *Z* value.

The Moran I is an extension of Pearson’s correlation coefficient to spatial neighbors and provides a score. If the score is positive and statistically significant, i.e., greater than 1.96 at a significance level of 5%, it can be concluded that the data exhibit positive spatial autocorrelation (areas of similar attributes). If the standardized value is negative and statistically significant, i.e., less than –1.96 at a significance level of 5%, it can be concluded that the data exhibit negative spatial autocorrelation (neighboring areas tend to have different attribute values). Finally, if the standardized value is within the interval [–1.96; 1.96], the null hypothesis or spatial randomness is accepted.

### Local spatial variation in food patterns

Although the Moran I reveals the existence of spatial autocorrelation for the data set, the Moran I does not allow detecting how the global pattern is distributed locally given that places may be observed with high spatial correlation for the variable or random correlations. This limitation is solved using the local method that can identify the characteristics of the clusters in terms of their location, size and magnitude. The Moran Local Index or LISA can be calculated to identify “clusters” and spatial outliers as follows:

{I_j} = \,\,\frac{{\left( {{x_1} - x} \right)}}{{{m_{\it 2}}}}\,\,\,\,\,\sum\limits_{j = 1}^n {{w_{ij}}} \left( {{x_j} - x} \right)\,\,{\rm para}\,\,i\,\, \ne \,\,j

where *m* is the variance, *n* is the number of units in the map, *W_ij_* is the distance matrix that defines whether the units are neighbors, X_i_ is the value of the adherence to each of the patterns in each geodemographic unit and Z is the mean of the variable X.

The interpretation of the Moran Local Index or LISA is similar to the global statistic. The results are evaluated based on five scenarios: High-High, Low-Low, Low-High, High-Low and not significant. The LISAs were defined with the identification of departments with high and low scores as well as spatial outliers. Groups of departments with high scores are generally referred to as hot spots (High-High), whereas those with low scores are cold spots (Low-Low). A spatial outlier represents the place that exhibits a mix of high and low scores in the neighboring areas (Low-High, High-Low) [18,29].

### Graphs of the food transition phenomenon in Colombia

The analysis aimed to establish the spatial variation in food patterns in Colombia based on the three patterns identified by Ocampo et al. [6] and Herrán et al. [7]. To detect both global and local spatial clustering, GeoDa software [30] with a maximum spatial conglomerate size ≤ 5% of the total population (spatial analysis of clusters) was used. These results are shown in maps with shading used to denote areas as follows: dark black and hot for High-High spots, light gray and cold for Low-Low spots, and intermediate gray spots for high dispersion or random spatial distribution (Low-High, High-Low). The maps and databases were prepared by the authors.

## Results

We analyzed the information based on total adherence to the three food patterns as well as on sex in 33 departments or geodemographic units.

### Medium adherence to food patterns

The mean age in the geodemographic units was 37.2 years (95% CI: 33.8 to 39.8). The minimum age was 33.8 years in La Guajira, and the maximum was 39.8 years in Atlantico. The average value of adherence (Z) to a consumption pattern allows to establish when comparing the three patterns in a geodemographic unit, which is predominant. The same occurs when comparing the average value of adherence to the same consumption pattern by sex. An average value of 0.00, means that in the geodemographic unit the adherence is in the average, negative values, mean adhesion loss and positive, greater adherence relative to other geodemographic units. Despite the extreme values showing the loss of adherence to the traditional/starch pattern, as in the geodemographic unit San Andrés and Providencia, –0.87 (Z), or adherence values showing its predominance, as in the geodemographic unit Caldas, 0.58 (Z) (Table 2), the average adherence to the traditional/starch food pattern was 0.00 (95% CI: –0.12 to 0.12). The value was 0.09 (95% CI: –0.06 to 0.23) in men and –0.07 (95% CI: –0.18 to –0.04) in women. There were no differences in the regions in adherence to the traditional/starch pattern according to sex: the mean difference was 0.16 (95% CI: –0.23 to 0.55). Additionally, adherence values were very similar by sex: Pearson’s *r* correlation between values by sex was 0.88 (95% CI: 0.77 to 0.94). The mean adherence to the fruit-vegetable/dairy food pattern was –0.07 (95% CI: –0.16 to 0.03). The value was –0.17 (95% CI: –0.26 to –0.09) in men and 0.01 (95% CI: –0.09 to 0.12) in women. The mean difference was –0.19 (95% CI: –0.47 to 0.09), and Pearson’s *r* correlation between values by sex was 0.89 (95% CI: 0.78 to 0.94). The average adherence to the snack food pattern was –0.03 (95% CI: –0.11 to 0.05). This value was –0.03 (95% CI: –0.06 to 0.12) in men and –0.07 (95% CI: –0.15 to 0.01) in women. The mean difference was –0.01 (95% CI: –0.18 to 0.38), and Pearson’s *r* correlation between values by sex was 0.83 (95% CI: 0.68 to 0.91). Adherence values based on pattern, sex and geodemographic units are presented in Table 2 and Table S1 (*supplementary material available online*).

**Table 2:** Score (Z) of adherence for three patterns of food consumption in the adult population (18–64 years) according to the geodemographic unit. Colombia, 2010.

Unit Geodemographic	Population	Adherence score (Z)^a^		
		Fruit/Vegetable/Dairy	Traditional/Starch	Snack

Antioquia	3784595	0.02	0.28	0.24
Atlántico	1413864	0.09	–0.48	0.07
Bogotá. D.C.	4770513	0.41	–0.25	0.14
Bolívar	1132004	0.25	–0.30	0.22
Boyacá	714063	0.06	–0.05	–0.16
Caldas	593766	–0.10	0.58	–0.09
Caquetá	242861	–0.36	0.36	–0.20
Cauca	749288	–0.26	0.13	–0.30
Cesar	533900	0.03	–0.13	0.07
Córdoba	890482	0.14	–0.12	–0.10
Cundinamarca	1480268	0.18	0.07	0.03
Chocó	235469	–0.30	–0.20	0.23
Huila	615128	–0.06	0.37	–0.30
La Guajira	441303	–0.10	–0.40	0.22
Magdalena	644717	0.03	–0.29	0.16
Meta	520921	0.11	0.06	–0.16
Nariño	945959	–0.13	0.26	–0.27
Norte de Santander	746840	–0.05	0.01	–0.04
Quindío	335038	–0.04	0.31	–0.10
Risaralda	567792	0.10	0.45	–0.15
Santander	1222400	0.07	0.20	–0.14
Sucre	456276	0.17	–0.32	–0.05
Tolima	790829	0.04	0.07	–0.20
Valle del Cauca	2739808	0.14	0.05	–0.14
Arauca	126804	0.12	0.04	0.02
Casanare	188607	–0.03	0.47	–0.09
Putumayo	173221	–0.43	0.37	–0.36
San Andrés. Providencia	46315	0.11	–0.87	0.76
Amazonas	36534	–0.65	–0.43	–0.01
Guainía	19590	–0.54	–0.22	–0.19
Guaviare	53412	–0.01	0.36	0.06
Vaupés	20476	–0.74	–0.59	0.19
Vichada	31308	–0.50	0.15	–0.25

^a^ Based on factor analysis.

### Global spatial variation in food patterns

For the three food patterns, the values for the Total Moran I for men and women were positive and significant (p < 0.05 for all). The Moran I was consistently increased in the fruit-vegetable/dairy pattern: 0.50 for the total population, 0.47 for men and 0.46 for women. The index was reduced for the snack pattern: 0.23 for the total population, 0.24 for men and 0.17 for women. These findings demonstrate the existence of well-defined clusters of food patterns in the geodemographic units studied. Table 3 presents the values achieved for the Moran I and its level of significance.

**Table 3:** Moran I Global Index for three patterns of food consumption in the geodemographic units of Colombia.

Food Pattern	Moran Global Index (I)^a^			P Value^b^		

	Total	Males	Females	Total	Males	Females

Fruit/Vegetable/Dairy	0.50333	0.47200	0.45637	0.005	0.003	0.009
Snack	0.22949	0.24154	0.17337	0.072	0.044	0.087
Traditional/Starch	0.25074	0.28786	0.21834	0.062	0.047	0.084

^a^ Based on 33 geodemographic units. ^b^ Based on ANOVA.

### Local spatial variation in food patterns

In the traditional/starch and fruit-vegetable/dairy food patterns, significant high variations were identified. These hot spots for the fruit-vegetable/dairy pattern were located in the geodemographic units of Bolívar, Sucre, Córdoba and Antioquia (the Northwestern region).

For the traditional/starch pattern, the hot spots were located in the geodemographic units of Meta, Cundinamarca, Tolima, Risaralda and Cauca (the Andean and South-Central regions). The significantly low variations or cold points for the fruit-vegetable/dairy pattern were located in the units of Guainía, Caquetá, Vaupés and the Amazon (the Southeastern Region). The cold spots for the snack pattern were located in the units of Tolima, Huila, Cauca, Nariño, and Putumayo (the Southwestern Region). A single geodemographic unit exhibited significantly low variation for the traditional/starch pattern: the department of Magdalena (North or Caribbean region).

The previous results from the assignment of patterns to geodemographic units based on spatial exploration and statistical significance are consistent with those generated from LISA. For the traditional/starch pattern, statistically significant clusters that are equivalent to hot spots were detected in the Meta, Tolima and Cauca units. In addition, for the traditional/fiber pattern, the same department of Magdalena was identified as a nonsignificant cluster or cold spot. For the fruit-vegetable/dairy pattern, the nonsignificant clusters were located in the geodemographic units of Caquetá and Vaupés. For the snack pattern, the nonsignificant clusters were located in the geodemographic units of Cauca and Nariño.

### Graphic location of the nutrition transition occurring in Colombia (Maps)

**Figure 1:**
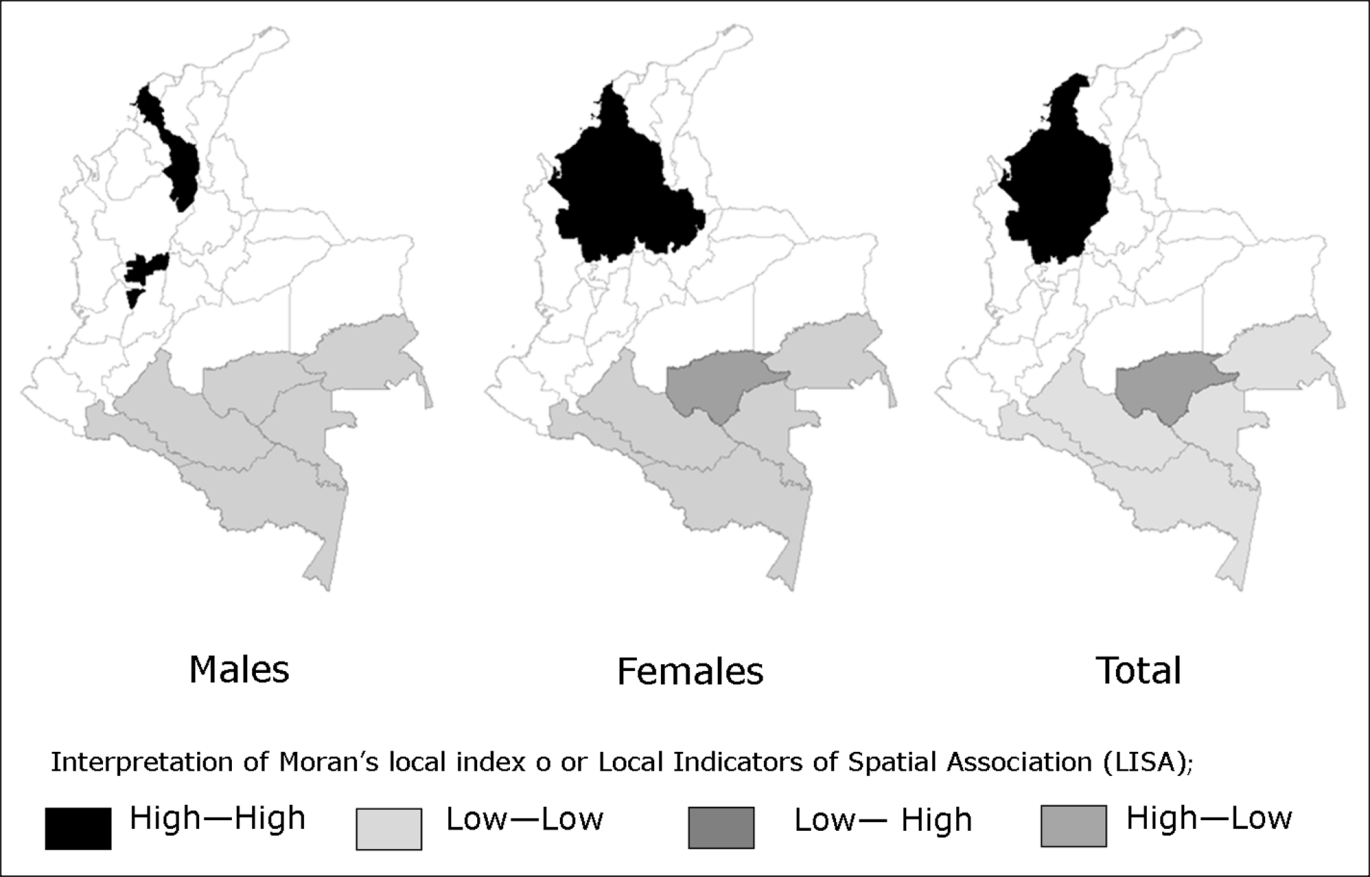
Cluster for the Fruit-Vegetable/Dairy Food Pattern: Food Transition. Colombia, 2010.

**Figure 2:**
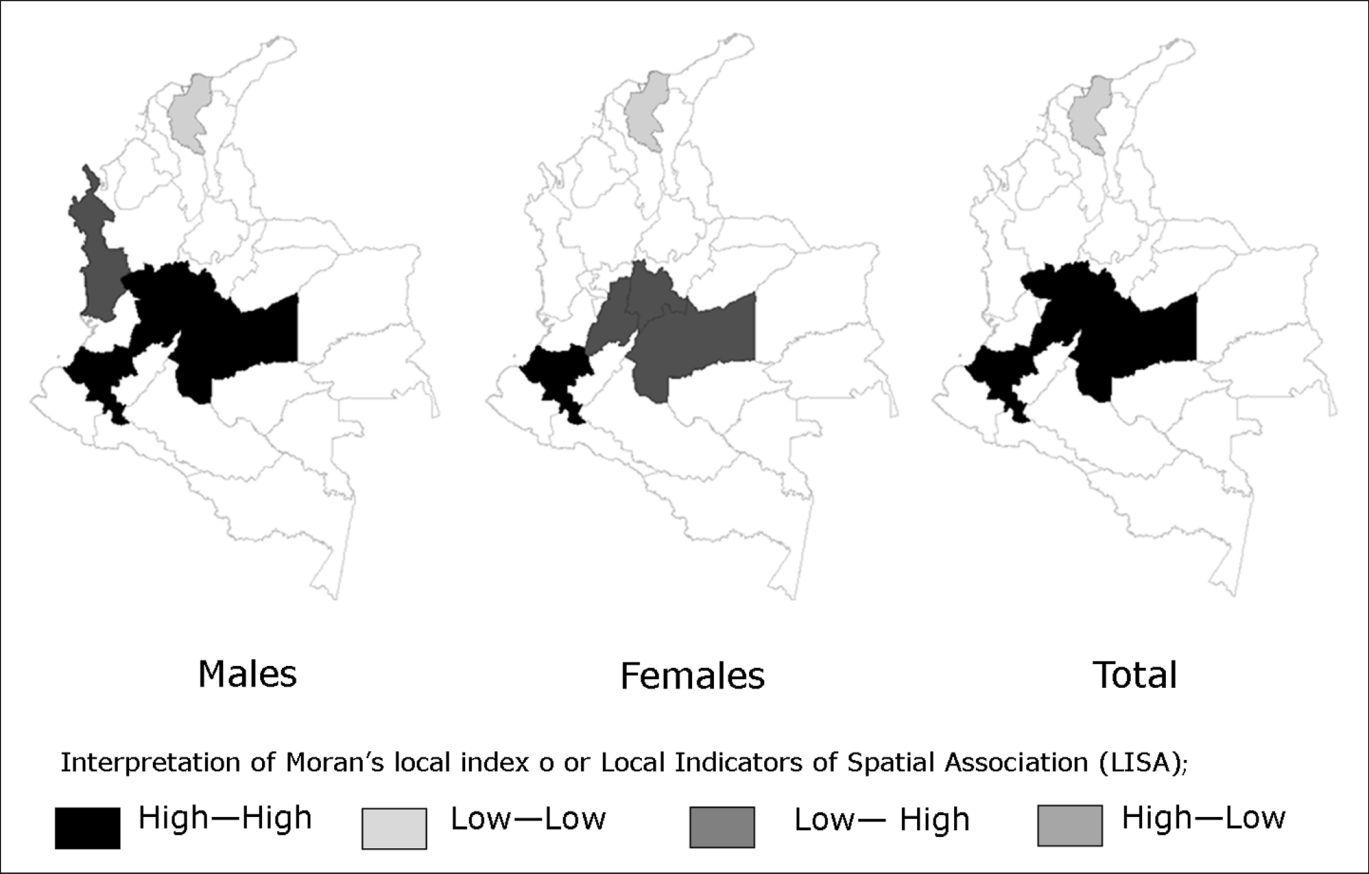
Cluster for the Traditional/Starch Food Pattern: Food Transition. Colombia, 2010.

**Figure 3:**
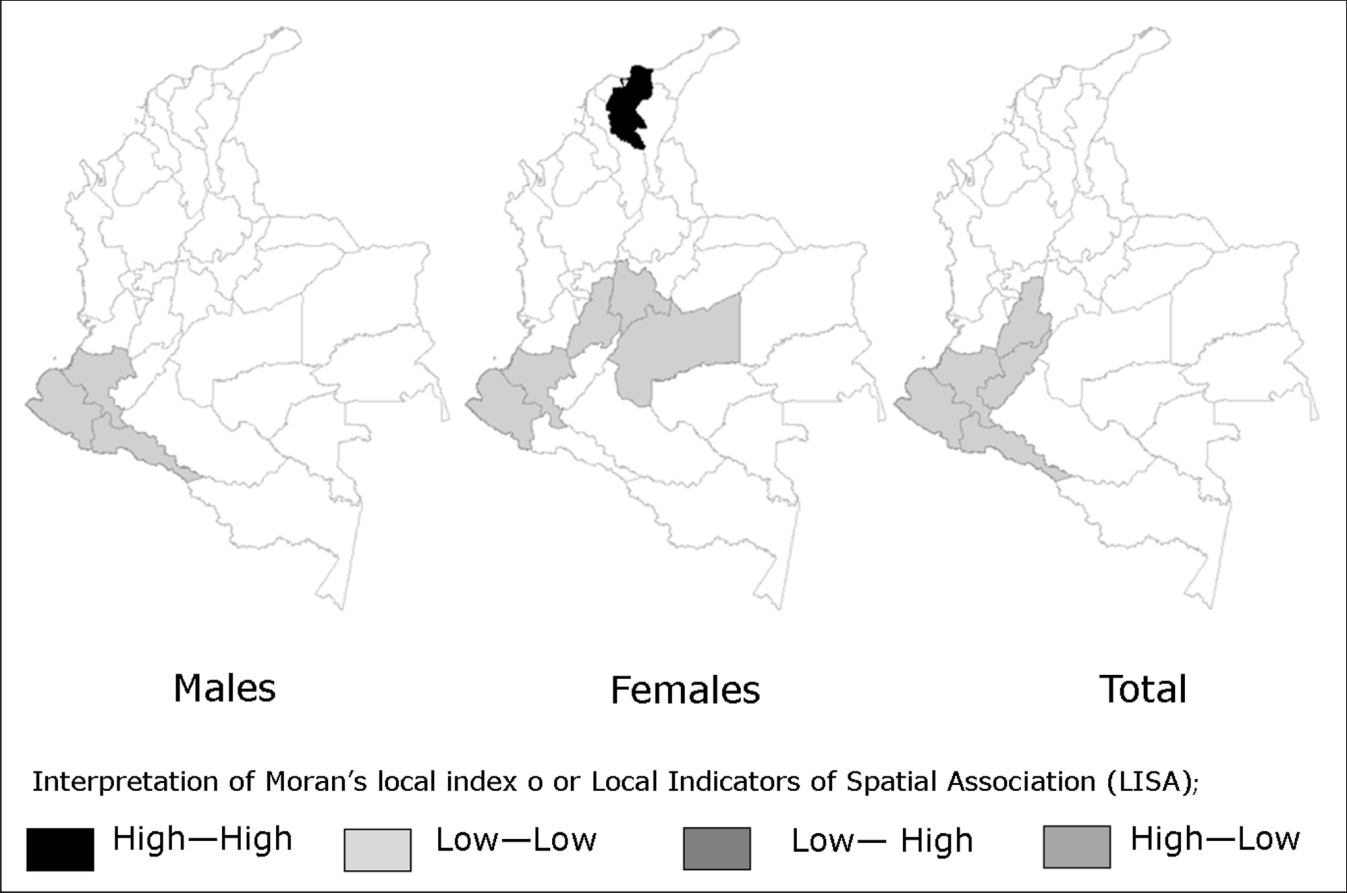
Cluster for the Snack Food Pattern: Food Transition. Colombia, 2010.

The results described above are graphically described in Figures 1, 2, 3.

## Discussion

This work is the first to investigate the spatial variation in food patterns in Colombia. The results cannot be compared because the food transition in Colombia was only recently declared and there is no history of this type of analysis. We found that the food transition occurring in Colombia is not homogeneous and that there are well-defined clusters of adherence to the three predefined food patterns. In the Northeast, the fruit-vegetable/dairy pattern predominates. In the Andean and South-Central regions, the traditional/starch pattern predominates. The snack pattern is strongly predominant in the department of Magdalena (Caribbean region) and lacks a significant presence in other regions of the country. The snack pattern is very weak where the traditional/starch pattern predominates. In addition, although spatial groupings are maintained in the macro region, there are marked differences by sex within these groupings. The finding that high or low adherence to food patterns are grouped significantly at a regional level reconfirms the existence of regional “food cultures”.

Food transition is a novel concept that emerged in countries where the traditional pattern still coexists with other patterns and is referred to in a particular manner based on the foods that compose these food patterns [6,7 10 12,13]. The Western diet or Western pattern emerged in developed societies where the traditional pattern had ceased to exist or was no longer predominant [9,10,11] and the concept of nutrition transition explained through food stages was eventually absorbed. Food patterns are associated with culture [16]. The fruit-vegetable/dairy pattern is part of the Antioquia and Black or fluvial mining cultural complexes, particularly in the zone of influence of the Cauca and Magdalena rivers and low valley. The traditional/starch pattern is spatially contained in the Andean or American cultural complex. It is interesting that the snack pattern has a very weak presence in the same spatial zone of the Andean cultural complex and predominantly in women in the department of Magdalena (Caribbean region). The traditional/starch pattern does not coexist with the snack pattern and vice versa.

The application of spatial analysis to explore the geographical distribution of food patterns has been limited mainly to the developed world [17,31]. However, the study of the spatial grouping of health-related factors is not completely new. Previous spatial analyses in the field of public health have focused mainly on detecting differences in the prevalence of obesity and type 2 diabetes and the patterns of medical practice in the metropolitan census areas of the United States [32,33].

In Canada, this type of analysis has been used to reveal overweight and obese groups and highlight the importance of prevention strategies by geographic zones according to the specific needs of the population, with the objective of focusing public health resources [34]. In the United Kingdom, spatial analysis has been used to estimate the determinants of childhood obesity, identifying geographical variations where public health intervention programs could be specifically designed for communities living at high risk [35]. This analysis has also been used with data at the individual level to detect obesity groups and explain the data based on the composition of the neighborhood (geographical areas – obese genetic populations) and socioeconomic characteristics [36].

Unlike the aforementioned variables, namely, body weight, medical practices or diabetes, food practices are related to beliefs, perceptions and deep-rooted cultural values [16,37], which are intuitively closely related to the region in which one lives. This notion may be especially true for regions with a low “migration” rate, as noted in a considerable portion of the Colombian territory. A study conducted in the Netherlands is very similar to the present study and reaches similar conclusions [17]. Food patterns are associated with food cultures, and spatial analysis is useful to identify healthy and unhealthy patterns. In addition, this information is helpful to focus action in search of healthier diets. Of course, the context and patterns identified in the Dutch study are not comparable with those reported here.

The spatial grouping as an ecological explanation where culture is another explanatory element exhibits other important aspects, such as the economy (legal and illegal) and the symbolic valuation of goods, including food, by subjects in the territory. The Antioquia cultural complex has been and is considered the most economically developed in the country due to industry, agriculture, livestock and mining activities that are complemented by fishing activities in the lower valleys of the Cauca and Magdalena rivers. This ecological type association is consistent with what has already been widely described in developed countries. Specifically, the fruit-vegetable/dairy pattern predominates in areas of greater wealth or development. The American Andean cultural complex would explain the predominant adherence to the traditional/starch pattern because it contains peasants and farmers (with the exception of areas such as the capital Bogota) and the food practices of the indigenous peoples are still maintained (e.g., potato, corn, wheat, barley). In addition, the Andean region and especially the Southwest are relatively poor and marginal compared with the Northwest region, where the Antioquia complex is located. If we accept the food transition as a consequence of wealth and development, the spatial grouping fits perfectly with this theory.

### Scope and limitations of the study

The subsample that answered the FFQ in the ENSIN-2010 is representative of the Colombian population, so there is no possibility of selection bias. Incorporating the complex sampling design in all the analyses ensured the above. The FFQ is the most commonly used method in nutritional epidemiology to approximate usual consumption. The FFQ applied in the ENSIN-2010 was designed by experts to study nutritional problems in Colombia. Before applying it, the facial validity of all its items was ensured, and the response categories for food frequency have undergone validity and reproducibility studies in the Colombian population [23]. Having limited the analysis to a range of weight and height minimized the information bias in the FFQ. This study was conducted using cross-sectional data; therefore, the explanations given are based on spatial and statistical associations. Causal explanations are not possible. In addition, although the ENSIN-2010 is a national and representative survey, it did not cover the entire human landscape, which could make “clusters” in the non-sampled areas invisible. Strengths of the study include the following: a) data that are representative of the country and achieved in a national survey; b) the plausibility of the associations established between adherence to food patterns and cultural complexes and the level of wealth and development achieved in the regions; c) the meta-mathematical techniques developed and the power provided by grouping data from ecological studies; d) the identification of adhesion clustering in geographic regions and cultural complexes, which allows the design of interventions that are focused and consistent with the symbolic value of food and goods; and e) this clustering resulting from spatial analysis serves as the baseline for monitoring changes in food patterns based on future ENSIN.

## Conclusions

Three food patterns coexist in Colombia; however, each pattern predominates as a cluster in geographic areas and well-defined cultural complexes. The fruit-vegetable/dairy pattern predominates in the Antioquia and Black cultural complex and in the richest and most productive geographical region of the country. The traditional/starch pattern predominates in the Andean or American cultural complex and the Andean region, which, with the exception of the capital Bogota, is relatively poor compared with the Northwest and has been inhabited predominantly by peasants, farmers and indigenous peoples who have preserved their food traditions. The snack pattern is very weak in the same spatial zone as the traditional/starch pattern and very strong in the women of the department of Magdalena in the Caribbean region. The food transition occurring in Colombia is not homogeneous and is associated with the characteristics of geographical regions, such as culture, wealth and the level of development.

## Additional File

The additional file for this article can be found as follows:

10.5334/aogh.1643.s1 Table S1. Scores (Z) of adherence to three food patterns in the adult population (18–64 years) according to the geodemographic unit and sex. Colombia, 2010.
