# Supplementary material for: Food Changes and Geography: Dietary Transition in Colombia
Source: Ann Glob Health. 2019 Mar 5;85(1):28. doi: 10.5334/aogh.1643 (PMC6634597; doi:10.5334/aogh.1643)
Supplement: Table S1. — Scores (Z) of adherence to three food patterns in the adult population (18–64 years) according to the geodemographic unit and sex. Colombia, 2010. [file agh-85-1-1643-s1.pdf]

Table S1 Scores (Z) of adherence to three food patterns in the adult population (18-64 years) according to the geodemographic unit and sex. Colombia, 2010.

Unit Geodemographic	Adherence score (Z) [a]							
	Population		Fruit-Vegetable / Dairy		Traditional / Starch		Snack	
	M	F	M	F	M	F	M	F
Antioquia	1827885	1956710	-0.05	0.08	0.43	0.17	0.36	0.14
Atlántico	692121	721743	-0.04	0.19	-0.45	-0.50	0.22	-0.05
Bogotá. D.C.	2270072	2500441	0.33	0.47	-0.10	-0.37	0.34	-0.03
Bolívar	562782	569222	-0.02	0.42	-0.29	-0.31	0.27	0.19
Boyacá	356069	357994	-0.19	0.23	0.05	-0.11	-0.07	-0.23
Caldas	287483	306283	-0.16	-0.05	0.79	0.39	-0.01	-0.17
Caquetá	121011	121850	-0.44	-0.30	0.33	0.39	-0.12	-0.26
Cauca	380335	368953	-0.29	-0.24	0.10	0.16	-0.36	-0.25
Cesar	262030	271870	-0.03	0.09	-0.09	-0.17	0.06	0.09
Córdoba	442173	448309	0.01	0.25	-0.12	-0.13	-0.08	-0.11
Cundinamarca	735049	745219	0.15	0.21	0.31	-0.08	0.14	-0.04
Chocó	116432	119037	-0.40	-0.25	-0.21	-0.20	0.23	0.23
Huila	306913	308215	-0.19	0.03	0.54	0.22	-0.16	-0.41
La Guajira	214696	226607	-0.33	0.05	-0.42	-0.39	0.29	0.18
Magdalena	323262	321455	0.05	0.01	-0.19	-0.41	0.21	0.09
Meta	258859	262062	-0.05	0.22	0.22	-0.12	0.04	-0.30
Nariño	472800	473159	-0.29	-0.01	0.41	0.15	-0.32	-0.23
Norte de Santander	366455	380385	-0.28	0.11	0.15	-0.09	-0.02	-0.05
Quindío	162751	172287	-0.14	0.02	0.45	0.22	-0.02	-0.15
Risaralda	273230	294562	0.00	0.18	0.83	0.15	-0.08	-0.21
Santander	600627	621773	-0.09	0.24	0.33	0.05	-0.03	-0.26
Sucre	230681	225595	-0.02	0.35	-0.22	-0.41	0.02	-0.11
Tolima	393335	397494	0.03	0.04	0.36	-0.12	-0.21	-0.20
Valle del Cauca	1312013	1427795	0.04	0.23	0.14	-0.02	-0.07	-0.20
Arauca	63678	63126	0.07	0.16	0.20	-0.07	0.04	0.01
Casanare	95961	92646	-0.17	0.10	0.56	0.39	-0.06	-0.12
Putumayo	87860	85361	-0.47	-0.41	0.40	0.35	-0.36	-0.35
San Andrés. Providencia	22894	23421	-0.10	0.23	-0.83	-0.89	0.89	0.68
Amazonas	18508	18026	-0.70	-0.60	-0.48	-0.36	-0.13	0.13
Guainía	10210	9380	-0.61	-0.48	-0.34	-0.10	-0.22	-0.16
Guaviare	28793	24619	-0.23	0.18	0.58	0.18	0.25	-0.10
Vaupés	10443	10033	-0.70	-0.78	-0.60	-0.59	0.22	0.16

M Males. **F** Females. [a] Based on factor analysis.
